# A prospective study on tumour response assessment methods after neoadjuvant endocrine therapy in early oestrogen receptor-positive breast cancer

**DOI:** 10.1186/s13058-023-01756-8

**Published:** 2024-01-03

**Authors:** Joanna I. López-Velazco, Sara Manzano, María Otaño, Kepa Elorriaga, Núria Bultó, Julio Herrero, Ainhara Lahuerta, Virginia Segur, Isabel Álvarez-López, Maria M. Caffarel, Ander Urruticoechea

**Affiliations:** 1 Biogipuzkoa (Previously Known As Biodonostia) Health Research Institute, Paseo Dr Begiristain S/N, 20014 San Sebastian, Spain; 2 Gipuzkoa Cancer Unit, OSI Donostialdea - Onkologikoa Foundation, Paseo Dr Begiristain 121, 20014 San Sebastian, Spain; 3 IKERBASQUE, Basque Foundation for Science, Bilbao, Spain

**Keywords:** Neoadjuvant endocrine therapy, Aromatase inhibitors, Tumour cellularity size, Oestrogen receptor (ER)-positive breast cancer, Pathological and radiological tumour response, Preoperative endocrine prognostic index (PEPI) score, Ki67

## Abstract

**Background:**

Neoadjuvant endocrine therapy (NET) in oestrogen receptor-positive (ER+) /HER2-negative (HER2-) breast cancer allows real-time evaluation of drug efficacy as well as investigation of the biological and molecular changes that occur after estrogenic deprivation. Clinical and pathological evaluation after NET may be used to obtain prognostic and predictive information of tumour response to decide adjuvant treatment. In this setting, clinical scales developed to evaluate response after neoadjuvant chemotherapy are not useful and there are not validated biomarkers to assess response to NET beyond Ki67 levels and preoperative endocrine prognostic index score (mPEPI).

**Methods:**

In this prospective study, we extensively analysed radiological (by ultrasound scan (USS) and magnetic resonance imaging (MRI)) and pathological tumour response of 104 postmenopausal patients with ER+ /HER2- resectable breast cancer, treated with NET for a mean of 7 months prior to surgery. We defined a new score, tumour cellularity size (TCS), calculated as the product of the residual tumour cellularity in the surgical specimen and the tumour pathological size.

**Results:**

Our results show that radiological evaluation of response to NET by both USS and MRI underestimates pathological tumour size (path-TS). Tumour size [mean (range); mm] was: path-TS 20 (0–80); radiological-TS by USS 9 (0–31); by MRI: 12 (0–60). Nevertheless, they support the use of MRI over USS to clinically assess radiological tumour response (rad-TR) due to the statistically significant association of rad-TR by MRI, but not USS, with Ki67 decrease (*p* = 0.002 and *p* = 0.3, respectively) and mPEPI score (*p* = 0.002 and *p* = 0.6, respectively). In addition, we propose that TCS could become a new tool to standardize response assessment to NET given its simplicity, reproducibility and its good correlation with existing biomarkers (such as ΔKi67, *p* = 0.001) and potential added value.

**Conclusion:**

Our findings shed light on the dynamics of tumour response to NET, challenge the paradigm of the ability of NET to decrease surgical volume and point to the utility of the TCS to quantify the scattered tumour response usually produced by endocrine therapy. In the future, these results should be validated in independent cohorts with associated survival data.

**Supplementary Information:**

The online version contains supplementary material available at 10.1186/s13058-023-01756-8.

## Introduction

Oestrogen receptor-positive (ER+)/human epidermal growth factor receptor 2-negative (HER2-) breast cancer (hereafter referred to as ER+ BC) represents almost 70% of all breast malignancies. Antiestrogenic or endocrine therapy is the cornerstone of ER+ BC treatment, although long-term resistance to this therapy (in the neoadjuvant or adjuvant setting) is a common event [1]. Neoadjuvant (or preoperative) endocrine therapy (NET) results in pathological and clinical response rates comparable to those observed with neoadjuvant chemotherapy (NCT), although with lower toxicity and decreased pathologic complete response (pCR) rates [2–5]. Three pioneer clinical trials (IMPACT, PROACT and P024) demonstrated that NET is effective in downsizing ER+ BC and facilitating breast-conserving surgery (BCS) and showed greater efficacy for aromatase inhibitors compared with tamoxifen [6–8]. As a consequence of these and other studies, NET, given for 4–8 months, is nowadays recommended by international guidelines for postmenopausal women presenting ER+ BC [9–11]. An important advantage of NET is that it allows “in vivo” evaluation of response, hence granting real-time examination of drug efficacy as well as investigation of the biological and molecular changes that occur after estrogenic deprivation. However, the lack of useful biomarkers of long-term efficacy of therapy has precluded the development of the neoadjuvant strategy for endocrine therapies.

In the management of patients under neoadjuvant systemic therapy (either NET or NCT), two important evaluations are performed. First, a preoperative assessment of radiological tumour response (rad-TR) determines the response grade and establishes the surgical strategy [12–14]. Next, surgical specimens are histopathologically evaluated to obtain prognostic information according to pathological tumour response (path-TR) scales [12, 13]. In the case of BC patients treated with NCT, there are well-stablished parameters to measure tumour response, such as RECIST criteria, Miller & Payne and Sataloff grading scales, and residual cancer burden (RCB) value [15–18]. Nowadays, only Preoperative Endocrine Therapy Prognostic Index (PEPI) score and Ki67 levels have been validated as prognostic markers after NET [4, 19–22]. Hence, tumours that show substantial down-staging after NET and present low Ki67 levels and PEPI score at surgery have an excellent long-term prognosis even without chemotherapy [1, 14, 19, 20]. However, they are not optimal and they are not routinely used due to, among other reasons, a lack of Ki67 measurement standardization [23, 24]. Importantly, these two biomarkers are not independent as PEPI score includes Ki67 levels [21]. In addition, novel potential prognostic and/or predictive biomarkers in the field of NET have been recently suggested [25]. For example, current clinical guidelines make contradictory recommendations about performing gene expression-based assays such as Oncotype, EndoPredict or PAM50 to select NCT or NET for patients with ER+ BC [11, 26]. Moreover, some studies have shown different response to neoadjuvant therapies (mainly NCT) between HER2-Low and HER2-0 ER+ breast tumours [27, 28].

In contrast to what happens for NCT, pCR after NET is a rare event and is not a useful marker of prognosis given its low likelihood [3–5]. In fact, previous studies suggest that ER+ BC tumours after neoadjuvant systemic therapy present a “diffuse cell loss” response at pathological level, which is characterized by a distribution of the tumour in multiple scattered foci or small groups of tumour cells without affecting overall tumour size [29]. Understanding the impact of tumour response to NET on long-term outcomes will help clinicians to individualize adjuvant treatment for ER+ BC in clinical practice.

In this context, there is an urgent need for the identification of robust, reproducible biomarkers of response to NET with long-term prognostic value. Ideally, these new biomarkers should be candidates for initial validation in retrospective series. In addition, the mentioned diffuse cell loss in ER+ BC patients treated with NET needs to be better characterized. In order to investigate the dynamics of tumour response, we generated a prospectively collected series of ER+ BC patients treated with NET. We characterized and compared tumour response by ultrasound scan (USS) and/or magnetic resonance imaging (MRI) with pathological tumour size (path-TS). Finally, we described a new biomarker with potential prognostic implications, called tumour cellularity size (TCS), which could help to characterize the response to NET in ER+ BC by an estimation of the diffuse cell loss.

## Methods

### Study population

We analysed clinical data from a cohort of patients treated in our institution between 2005 and 2019. Data were prospectively collected and retrospectively analysed. All were postmenopausal women with histologically confirmed, untreated, invasive, operable, larger than 10 mm and amenable for radiological follow-up, ER+ /HER2- non-metastatic BC. Patients had to be treated for at least 3 months with NET prior to surgery with curative intention. Patients were treated with aromatase inhibitors, unless contraindicated. Informed consent was obtained from all patients.

### Imaging and histopathological analysis

Tumour baseline assessment was performed by breast USS and/or MRI. Clinical response by USS was evaluated after 2 months of treatment and repeated every 2 months. MRI and/or USS were also performed before surgery to evaluate radiological tumour response (rad-TR). Surgical breast specimens were evaluated by the pathologist to determine pathological tumour response (path-TR), tumour size (path-TS) and residual tumour cellularity (%). Clinical (assessed by MRI and USS) or pathological tumour size corresponds to the major diameter of the tumour in millimetres (mm) and T-stage of the primary tumour was defined according to AJCC Cancer Staging Manual [30].

Immunohistochemistry analyses were performed in baseline formalin-fixed paraffin-embedded biopsies and surgical specimens to determine the expression of ER, progesterone receptor (PgR) and Ki67 levels, using international standards [31, 32]. ER, PgR and Ki67 were recorded as continuous variables. Ki67 score was defined as the percentage of tumour cells with Ki67-positive nuclear staining. At least 1000 tumour nuclei were counted per sample, according to the recommendations of Dowsett et al. [31]. The change in Ki67 (∆Ki67) after NET was calculated using the following equation: ∆Ki67 = [(Ki67 (%) in surgery specimen)—(Ki67 (%) in baseline biopsy)]/(Ki67 (%) in baseline biopsy). ∆Ki67 results were categorized into three groups depending on their magnitude of change. ∆Ki67 = − 1 means that Ki67 changes to zero in the surgery specimen. ∆Ki67 =  > − 1 to < 0 means that the tumour presented a decrease in Ki67 expression. Finally, ∆Ki67 ≥ 0 means that the tumour did not present any change in Ki67 expression or that Ki67 expression in the surgery specimen was greater than the one on baseline biopsy.

Rad-TR was defined using mRECIST 1.1 criteria [15]. According with this criteria, complete responses (CR) were defined as tumour disappearance and partial responses (PR) were defined as the reduction of the tumour diameter by ≥ 30%. An increase ≥ 20% in tumour diameter was qualified as progressive disease (PD). The rest of situations were qualified as stable disease (SD).

Path-TR was quantified using a modified Miller and Payne grading scale [16, 33]. In this scale, response grades 1 and 2 (no change or less than 30% loss of tumour cells, respectively) were regarded as SD. Grades 3 and 4 (reduction in tumour cells between 30 and 90% and > 90%, respectively) were considered as pathologic PR. Grade 5 (defined as no malignant cells identifiable in the tumour niche) was considered pathologic CR (pCR). In binary analyses, path-TR was defined as loss of tumour cells ≥ 30% (grades 3–5) and no path-TR as < 30% (grades 1–2).

Modified PEPI (mPEPI) score was determined on the basis of tumour characteristics of surgical specimen (i.e. tumour size, nodal involvement status and Ki67 staining), as previously published [21, 22]. Patients were classified into 3 mPEPI risk groups (I = 0, II = 1–3 and III = 4 +).

TCS, the novel score we introduce in this study**,** was calculated as the product of tumour cellularity in the surgical sample (%) and tumour diameter (path-TS, in mm).

### Statistical analyses

Statistical analyses were performed using GraphPad Prism version 9. For the descriptive statistical analyses, minimum, maximum and mean values were used. For Gaussians distributions, paired Student’s *t*-test was used to compare differences between two groups. For non-Gaussian distributions, Wilcoxon matched-pairs or Kruskal–Wallis tests were performed. Chi-square or Fisher’s tests were used to determine differences between expected frequencies. Spearman’s *r* coefficient (rho) for analyses, were used to quantify correlations (both with a 95% of confidence interval). *P* values < 0.05 were considered statistically significant. Unless otherwise specified, histograms represent mean values ± standard error of the mean (SEM).

## Results

### Tumour characteristics and change in tumour biomarkers after NET

A total of 104 patients with early ER+ /HER2- breast cancer were included in our study. The study population presented a mean age at diagnosis of 69 (47–93) years, and the mean NET duration before surgery was 7 months (3–39). The mean tumour size was 25 mm (10–90) assessed by MRI and 18 mm (7–40) by USS. The main administered NET drug was letrozole (*n* = 100), but some patients also received anastrozole (*n* = 2), tamoxifen (*n* = 1), or exemestane (*n* = 1). One patient was diagnosed with bilateral disease, and her two tumours were independently considered in our analyses. The principal characteristics of the tumours and their surgical management as well as the pathological changes after NET are summarized in Table 1. No significant decrease of histological grade was observed after NET (*p* = 0.12). Ki67, ER and PgR expression was assessed in all tumours pre- and post-NET treatment. NET significantly decreased all these three parameters, being the changes in Ki67 and PgR the most significant (both *p* < 0.0001, Table 1 and Additional file 1: Figure S1). While only 13 patients (12%) were cN + before treatment, 26 patients (25%) were pN + at pathological assessment. Regarding pathological tumour response to NET, only one case of pCR was recorded and most cases (72%) showed partial path-TR (Table 2). Most patients (81%) fell into low (I, *n* = 34, 35%) and intermediate (II, *n* = 45, 46%) mPEPI risk groups.

**Table 1:** Histopathological information and surgical management of tumours included in our series

Characteristics	Before NET	After NET
	(*n* = 105*)	(*n* = 104ª)
Histological grade [*n* (%)]		
I	23 (22)	34 (33)
II	78 (74)	69 (66)
III	4 (4)	1 (1)
Histological type [*n* (%)]		
Ductal	86 (82)	85 (82)
Lobular	10 (10)	12 (12)
Other special type	9 (8)	7 (6)
c/yp axillary node status [*n* (%)]		
Negative	91 (87)	72 (69)
Positive	13 (12)	26 (25)
N/A	1 (1)	7 (6)
Positive cells (%) [mean (range)]		
Ki67	20 (3–60)	9 (0–75)
Oestrogen receptor	94 (20–100)	90 (0–99)
Progesterone receptor	63 (1–100)	15 (0–99)
Breast surgery performed [*n* (%)]*		
BCS		95 (90)
Modified radical mastectomy		10 (10)

^*^One patient was diagnosed with bilateral disease and her two tumours were independently considered in the histopathological analysis and in its surgical management

ªOne tumour was not evaluable for biological characteristics at surgery because the patient achieved a pCR

c/yp axillary node status was determined clinically and pathologically before and after NET, respectively. *BCS* breast-conserving surgery. *N/A* not available

**Table 2:** Radiological (rad-) and pathological (path-) tumour response (TR) after NET

Rad-TR type [*n* (%)]	MRI	USS	Path-TR [*n* (%)]
N/A	7 (7)	14 (13)	0 (0)
Complete	27 (26)	16 (15)	1 (1)
Partial	40 (38)	49 (47)	76 (72)
Stable disease	29 (27)	24 (23)	28 (27)
Progressive disease	2 (2)	2 (2)	0 (0)

Rad-TR was evaluated by mRECIST 1.1 criteria, and path-TR was measured using a modified Miller and Payne grading scale. *N/A* not available

### Radiological examination of tumour size after NET underestimates pathological tumour size

**Fig. 1:**
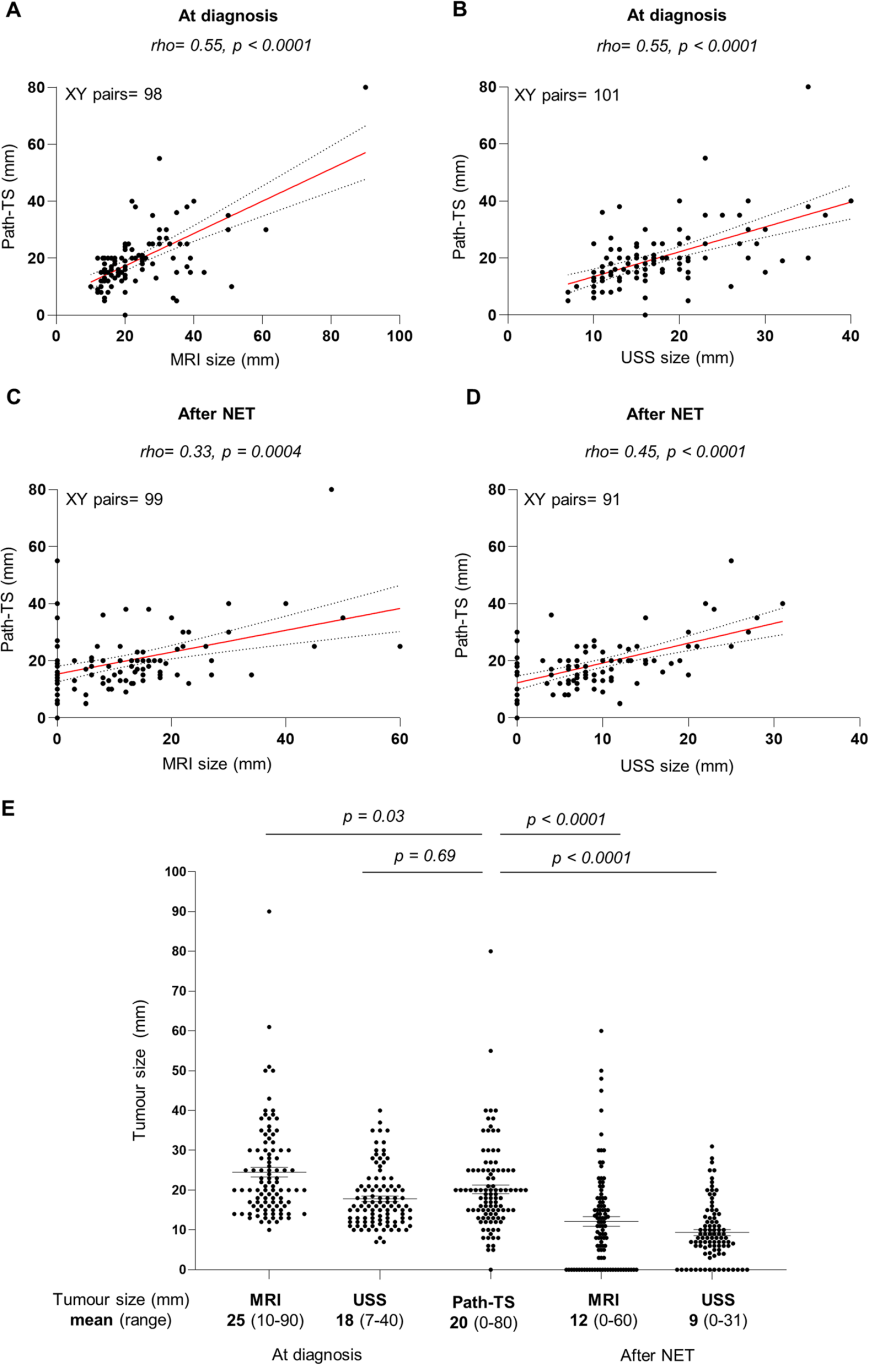
Comparison between pathological and radiological tumour size before and after neoadjuvant endocrine treatment (NET). (**A**–**D**) Correlation of pathological tumour size (path-TS) with MRI (**A** and **C**) and USS (**B** and **D**) measurements at diagnosis (**A**–**B**) and after NET (**C**–**D**). Spearman correlation coefficients (rho) and *p* values are shown. (**E**) Comparison of radiological tumour size (assessed by MRI and USS at diagnosis and after NET) with path-TS. *p* values were calculated using Kruskal–Wallis test

To determine which is the best radiological technique to predict pathological tumour size (path-TS) after NET, we compared tumour size measured by MRI and USS before and after treatment. As expected, radiological tumour size (rad-TS), measured by MRI or USS, both at diagnosis and after NET (just before surgery), significantly correlated with path-TS (Fig. 1A–D). Surprisingly, our results showed that path-TS correlated better with tumour size assessed by MRI and USS at diagnosis than after NET (Fig. 1A). To better visualize if the radiological evaluation before surgery precisely assesses tumour size after NET, we compared the mean value of tumour size assessed by each radiological technique, before and after NET, and by path-TS. As shown in Fig. 1E, MRI/USS measurements after NET were significantly lower than path-TS and, interestingly, radiological measures at diagnosis were more similar to path-TS than the measures after treatment. Actually, MRI and USS before surgery underestimated path-TS in 77% (76/99) and 92% (84/91) of the cases, respectively.

**Fig. 2:**
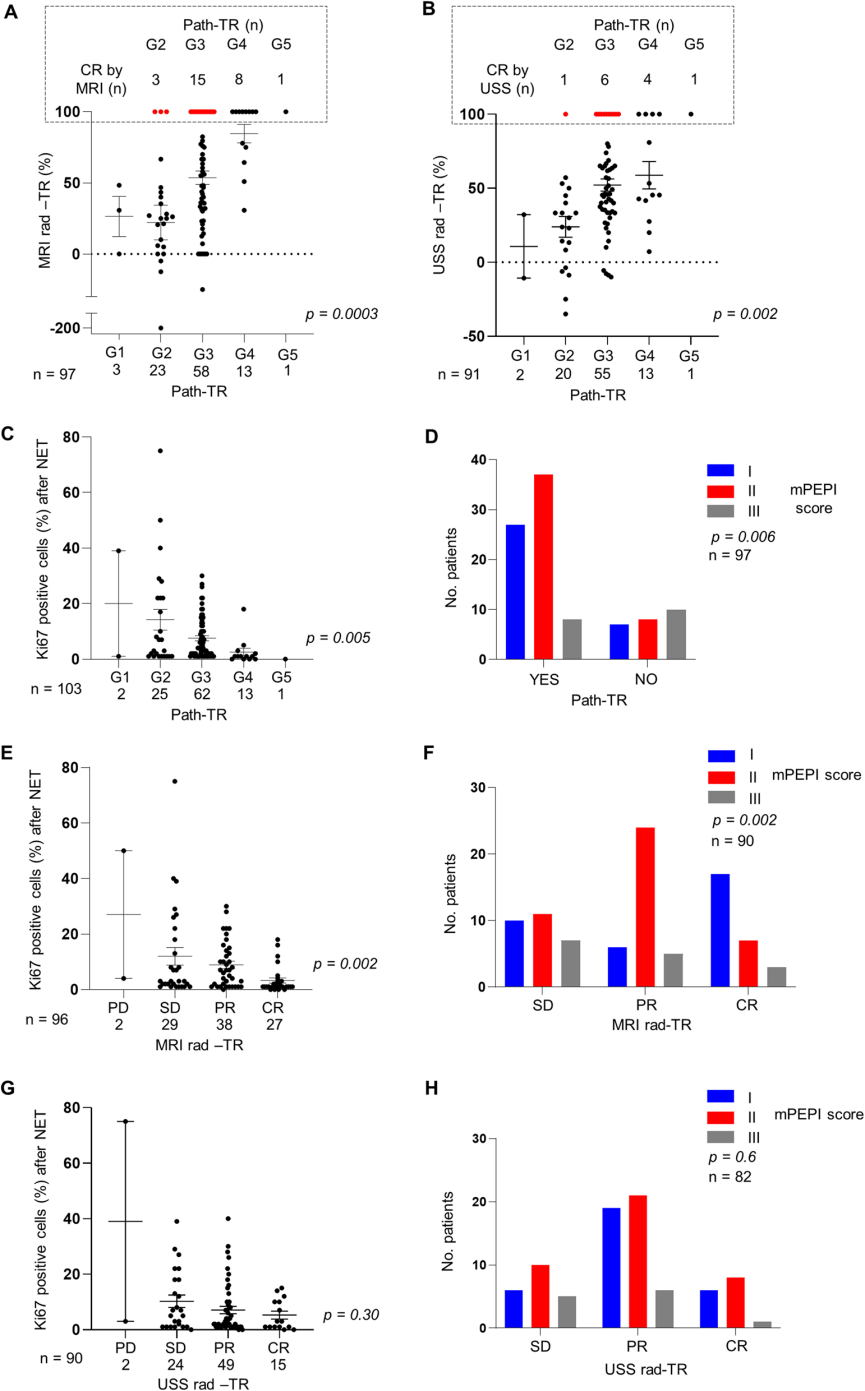
Evaluation of radiological tumour response (rad-TR) after NET by comparison with pathological tumour response (path-TR) and prognostic biomarkers for NET. (**A**–**B**) Rad-TR was assessed by MRI (**A**) and USS (**B**) and evaluated by mRECIST 1.1 criteria. Path-TR was evaluated using a modified Miller and Payne grading scale. In the upper square, tumours presenting complete response by MRI (**A**) or USS (**B**), but partial response (G2-G3) by path-TR, are highlighted in red. (**C**, **E** and **G**) Analysis of Ki67 levels at surgery in tumours classified according to their path-TR (**C**) and rad-TR by MRI (**E**) or USS (**G**). (**D**, **F** and **H**) Contingency analyses of the association between modified PEPI (mPEPI) score and path-TR (**D**) and rad-TR by MRI (**F**) or USS (**H**). *p* values were calculated using Kruskal–Wallis test (**A**–**C**, **E**, **G**) or Chi-square test (**D**, **F** and **H**). CR: complete response, PR: partial response, SD: stable disease, and PD: progression disease

Importantly, we also found that this disagreement in tumour size estimation by imaging and histopathological analysis affects the concordance between radiological (rad-TR) and pathological (path-TR) tumour response (Table 2). Complete rad-TR was observed in 27 (by MRI) and 16 (by USS) patients while only one patient presented a pCR by pathological assessment. To better visualize these discrepancies, we plotted the correlation between rad- and path-TR. As shown in Fig. 2A, B, we found that rad-TR assessed by MRI correlated better with path-TR than rad-TR assessed by USS, although both associations were statistically significant. Interestingly, we observed that a considerable number of tumours presented a complete (100%) rad-TR after NET but presented a low path-TR (G2 or G3, highlighted in red in Fig. 2A, B).

Next, we evaluated the association between rad- and path-TR with the two most accepted prognostic markers after NET: Ki67 levels and mPEPI score [14, 21, 22]. As expected, pathological responders presented significantly lower Ki67 levels at surgery and mPEPI score (Fig. 2C, D). Regarding rad-TR, both prognostic markers were associated with tumour response assessed by MRI (Fig. 2E, F), but, in the case of USS, there was not association between tumour response and Ki67 and mPEPI score (Fig. 2G, H).

In summary, our data support that radiological evaluation of tumour size after NET underestimates Path-TS and indicate that MRI could be more reliable than USS to assess response to NET.

### Tumour cellularity size is a new parameter to standardize the assessment of residual tumour content after NET

**Fig. 3:**
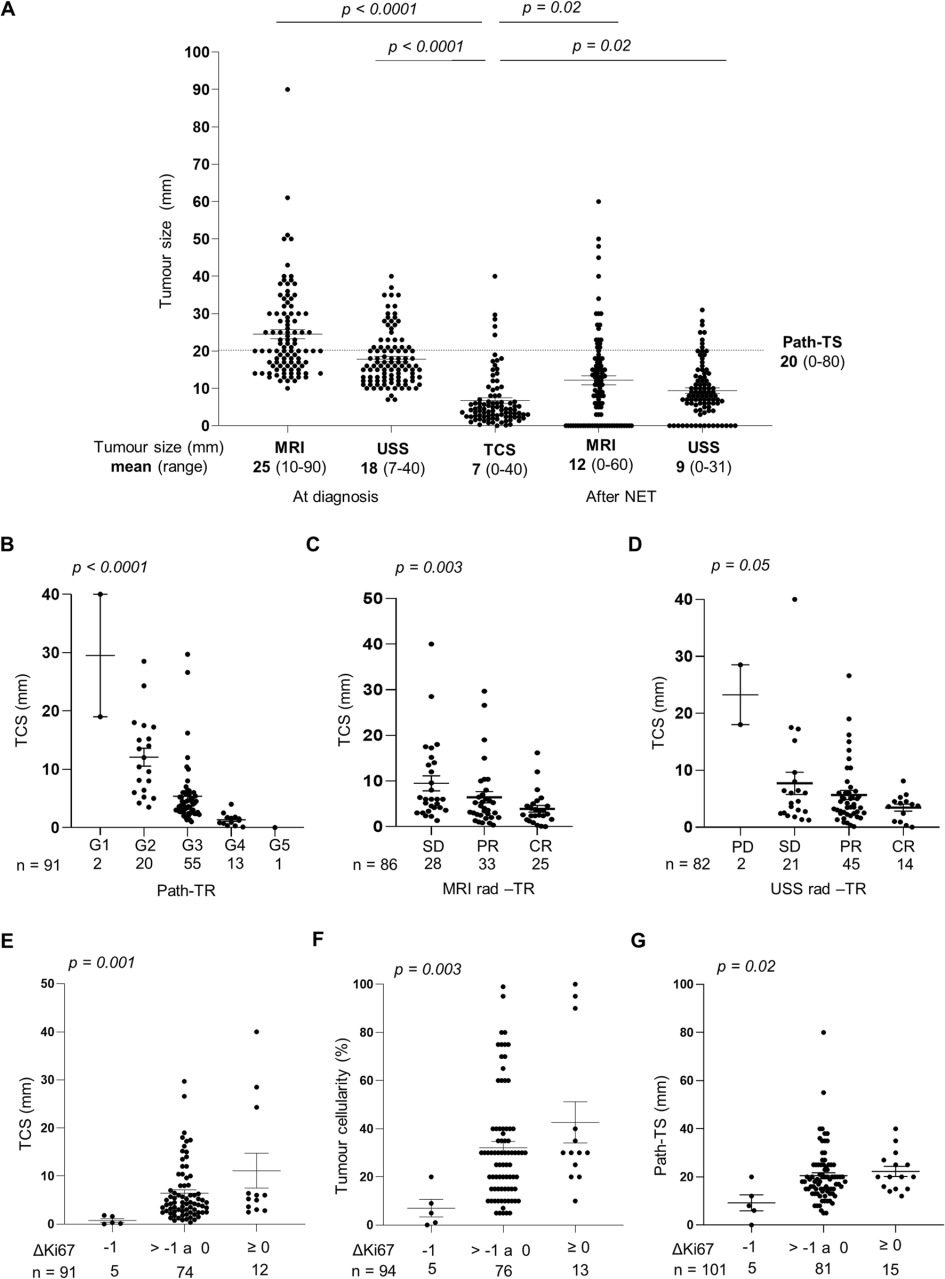
Tumour cellularity size (TCS) as a new parameter to measure response to NET. (**A**) Comparison of radiological tumour size (assessed by MRI and USS, before and after NET) with TCS. The dotted line indicates the mean of path-TS obtained for those tumours. (**B**–**D**) Association between TCS and pathological (path-TR) (**B**) and radiological response assessed by MRI (**C**) or USS (**D**). (**E**–**G**) Association between TCS (**E**), tumour cellularity (**F**) and pathological tumour size (path-TS) (**G**) with changes in Ki67 (∆Ki67) after NET. *p* values were calculated using Kruskal–Wallis test

Diffuse cell loss has been observed as a common pattern of tumour response after neoadjuvant therapies in ER+ (luminal) tumours [29]. In an attempt to better assess tumour response after NET, we propose a novel parameter called tumour cellularity size (TCS). TCS is the product of tumour cellularity (%) and tumour diameter (path-TS, in mm), which are routinely assessed in the clinical practice, and estimates the volume of remaining cells in the tumour bed after NET. First, we evaluated how TCS relates to radiological tumour size and response (Fig. 3). As seen in Fig. 3A, TCS values were much lower than path-TS and more similar to MRI or USS measures after NET compared to rad-TS at diagnosis or path-TS. We then analysed how TCS associates with radiological and pathological response (Fig. 3B–D). Our results showed that TCS inversely correlated with path-TR and with MRI rad-TR (Fig. 3B, C). However, the association between TCS and rad-TR determined by USS was not significant (Fig. 3D), in line with previous results supporting that MRI may be more adequate than USS to quantify response to NET. Taken together, our data indicate that TCS can quantify the tumour “diffuse cell loss’’ response observed in ER+ BC tumours after NET, and may capture better the biological response of those tumours and explain why the radiological pre-operative assessment of tumour size underestimates the path-TS.

In order to further evaluate if TCS can be used as a biomarker of response and prognosis for patients undergoing NET, we evaluated its association with changes in Ki67 (∆Ki67) (Fig. 3E–G) and Ki67 levels at surgery (Additional file 1: Figure S2), well-established prognostic markers after NET. We observed that ∆Ki67 and Ki67 expression at surgery correlated better with TCS than with tumour cellularity or path-TS (Fig. 3E–G, and Additional file 1: Figure S2). Consequently, tumours with high residual Ki67 expression (∆Ki67 > 0 and high Ki67 expression at surgery) also present a high TCS, suggesting that TCS could be a promising biomarker of response to NET.

**Fig. 4:**
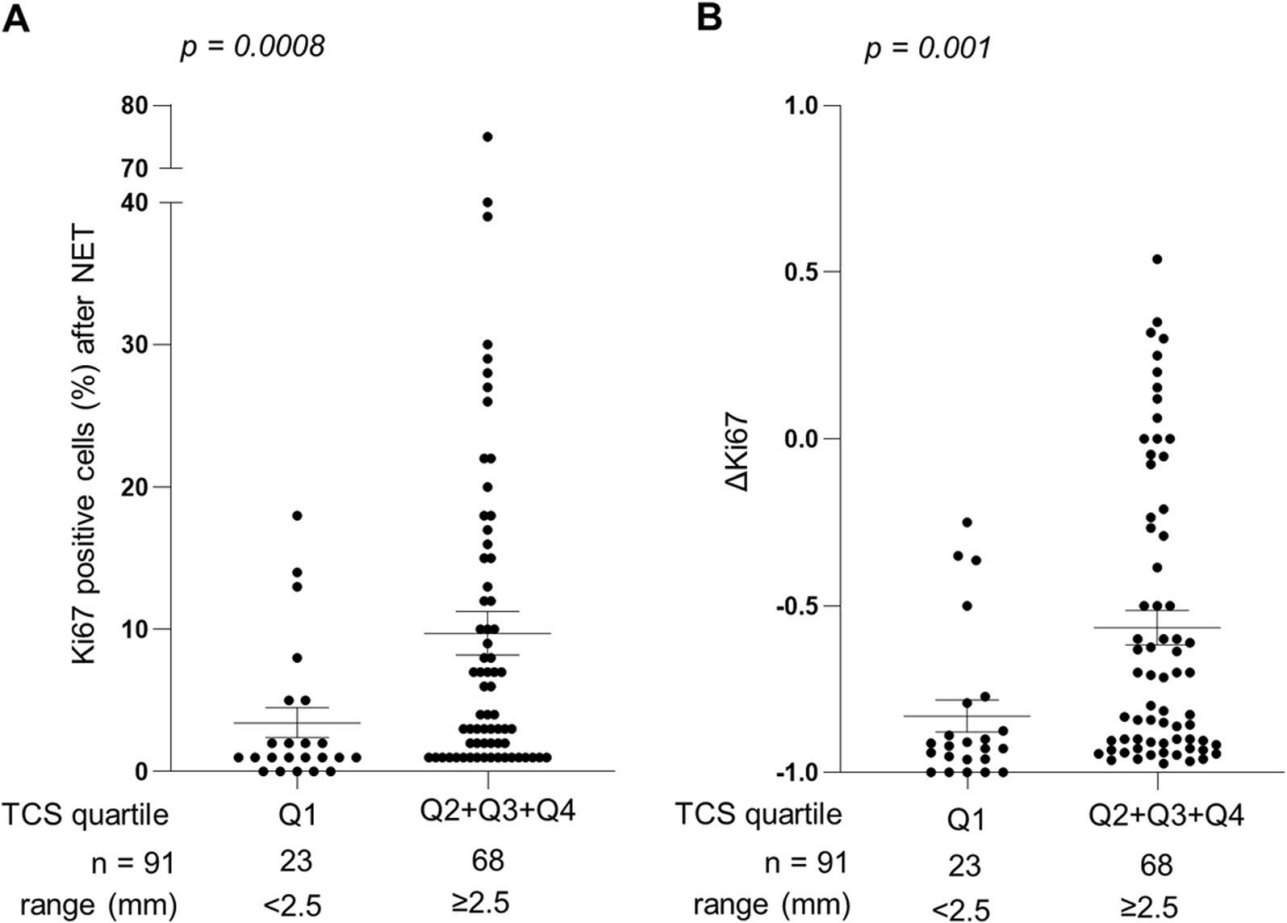
Identification of a tumour cellularity size (TCS) cut-off value to discriminate patients according to their response to NET. (**A**–**B**) Ki67 levels at surgery (**A**) and ∆Ki67 (**B**) in tumours with low (quartile 1, Q1) versus high (Q2, Q3 and Q4) TCS. *p* values were calculated using Mann Whitney test

Finally, to identify an initial cut-off value for which TCS can divide patients responding to NET from no responder patients, we analysed the relationship of TCS quartiles with Ki67 (Fig. 4 and Additional file 1: Figure S3). As mentioned before, TCS is positively correlated with Ki67 at surgery and ∆Ki67 (Additional file 1: Figure S3). Tumours with TCS < 2.5 mm (Q1) showed significantly lower Ki67 levels at surgery and ∆Ki67 compared with tumours with TCS ≥ 2.5 mm (Q2, Q3 and Q4, Fig. 4), suggesting that a TCS value < 2.5 mm could be used as a good cut-off value to identify patients responding to NET.

## Discussion

There are different reasons why neoadjuvant endocrine therapy (NET) is a very promising and attractive therapeutic strategy for ER+ BC patients. First, it is less toxic than neoadjuvant chemotherapy albeit resulting in similar pathological and clinical response rates and, indeed, it is already recommended by international guidelines for postmenopausal women [3]. Finally, it represents an ideal scenario for clinical research as it allows real time investigation of drug efficacy and of the molecular and biological changes in tumours after endocrine treatment. This may lead to the identification of novel biomarkers of response and new therapeutic strategies. However, NET remains an underused tool for ER+ BC because monitoring response is challenging, among other reasons [3, 34]. Many NET clinical trials use the radiological response rate (by pre-operative evaluation with USS, mammography or MRI using RECIST 1.1 criteria) and improvement of BCS rates as a primary objective to demonstrate effectiveness [3, 10, 13, 14]. However, our results, obtained from a prospectively collected series of 104 ER+ BC patients treated with NET, prove that the pre-operative radiological evaluation after NET underestimates path-TS. Importantly, our cohort is similar to others previously analysed in terms of response rates [10, 12, 13]. Previous reports have also shown that radiological and clinical evaluation after NET underestimate the lesion size [12], although Reis and cols found this difference negligible [35]. Clinically, these discrepancies between radiological and pathological responses may have strong surgical implications for the definition of lesion area and our data suggest that the radiological evaluation of tumour size might not be the most precise way to plan the resection area. In fact, the AJCC recommends that imaging findings after NET, NCT and radiotherapy are not considered elements of initial clinical staging [30].

In addition, our data show that radiological complete responses almost never parallel pCR. In our series, 26% and 15% of patients showed complete radiological response by MRI and USS, respectively, but only 1 patient achieved pCR. In fact, pCR after NET is a rare event and only occurs in less than 1% of the cases [3]. Usually, residual disease is found even in very good responder tumours, in the form of microscopically scattered residual cancer nests in the tumour bed [35]. This scattered or diffuse cell loss response is also observed after neoadjuvant chemotherapy in ER+ tumours [29].

While pathological assessment considers the maximum area occupied by the tumour and does not capture this scattered response, radiological evaluation after NET may reflect the diffuse cell loss response. This could explain why rad-TS after NET did not reflect path-TS in the surgery specimens in our cohort. Moreover, it may highlight the importance of further research to clarify the paradigm of NET as a tool to decrease the surgical volume for ER+ BC, at least in cohorts composed of small and low-risk tumours, similar to the one that we analysed in this manuscript. The difference between radiological and pathological evaluation of neoadjuvant systemic treatment (NST) is less frequently observed in triple-negative and non-luminal HER2+ tumours, which tend to present a shrinkage or concentric response (also called tumour collapse) to NST [29].

Nevertheless, we should also take into consideration that the complete response assessed by MRI may capture biological events with potential prognostic/predictive value, including normalization of tumour vessels. Hence, the event of CR by MRI, even without pCR, may define a prognostic category that deserves further study.

Despite the discrepancies between radiological and pathological evaluation of tumour response to NET, clinical and radiological monitoring of tumour response during the course of NET is necessary to early detect disease progression. Our data indicate that MRI is preferable than USS to assess response to NET, as (1) path-TR correlated better with rad-TR assessed by MRI than by USS and (2) MRI rad-TR was significantly associated with reduction of Ki67 in the surgical specimen and lower mPEPI score, the two most accepted prognostic markers after NET [14, 21, 22]. This association was not statistically significant in the case of USS. Our results are in agreement with previous reports supporting the use of MRI as the most accurate tool among other methods (clinical examination, USS and mammography) to assess tumour response to NST in breast cancer [3, 36].

As mentioned above, the diffuse cell loss response observed in ER+ tumours as a response to NST represents a challenge to evaluate tumour response to NET. We hypothesize that the parameter tumour cellularity size (TCS), presented herein, can be used to assess the scattered response to NET as it estimates the multiple scattered foci of tumour cells in the tumour niche. TCS is the product of tumour cellularity (%) and tumour diameter (path-TS, in mm) in the post-treatment surgical sample. We found that TCS significantly associated with rad-TR evaluated with MRI. As previously discussed, only PEPI score and Ki67 expression under treatment are validated prognostic markers for NET [4, 19, 22]. Importantly, TCS correlated better with ∆Ki67 than the percentage of tumour cellularity in the post-treatment sample and the path-TS. This may indicate that reduction in Ki67 expression is related to the tumour cellularity content even when the path-TS does not change after NET. The association between TCS and mPEPI score could not be evaluated as they are not independent variables since both include path-TS in their calculation [21]. Of note, in the case of NCT, residual cancer burden (RCB) is a good parameter to evaluate prognosis [18]. Nevertheless, this parameter may not be useful to assess response to NET in early ER+ BC cohorts as ours, since 83% of our patients clustered in class II, hindering further analyses. RCB, in contrast to TCS, includes nodal status as a parameter and this is a clear disadvantage for its use in NET cohorts similar to the one presented in this manuscript, as 87% of our patients presented negative c/yp axillary node status (Table 1).

Although this study sheds light on important points related to NET administration that hinder clinical evaluation of this therapy, it also has some limitations that should be addressed in the future: (i) recent studies support the strong prognostic value of Ki67 values after 2–4 weeks of NET [20], but biopsies after 2–4 weeks of NET were not available in our study, so we could not assess the association between Ki67 at this time point with tumour response; (ii) due to the characteristics of our cohort (small and operable tumours and majority of patients with low-risk and no candidates for mastectomy), we cannot properly evaluate if NET increases BCS rates, as described by others [6, 7]; (iii) our results should be validated in independent cohorts of ER+ BC with similar characteristics from different hospitals to discard biased results (e.g. the experience of each radiological facility may affect the precision of MRI and USS measurements); (iv) the power of TCS as a new biomarker to predict response to NET should be validated in independent cohorts with associated survival data.

## Conclusions

In summary, our results shed light on two clinically relevant and unanswered questions in the context of NET highlighted by Sella and cols [3]. One of them is which is the optimal imaging technique to pre-surgically evaluate residual disease after NET. Our data support the use of MRI over USS, but also prove that both imaging techniques underestimate pathological tumour size. As mentioned, this points to careful consideration of clinical and radiological TR to define the surgical resection tumour area and challenge the paradigm of the reduction of surgical volume by NET given that the initial radiological assessment seems to be the best value to define the tumour area even after therapy. The second unanswered question is the need of novel biomarkers to assess pathological response to NET. We propose a new biomarker called tumour cellularity size (TCS) that could be a promising candidate to use in combination with changes in Ki67.

### Supplementary Information

**Additional file 1. Figure S1.** Changes in tumour levels of: Ki67 (**A**), Oestrogen receptor (**B**) and Progesterone receptor (**C**) before and after NET. **Figure S2.** Association between tumour cellularity size (**A**), tumour cellularity (**B**) and pathological tumour size (**C**) with Ki67 levels at surgery. Spearman correlation coefficients (rho) and *p* values are shown. **Figure S3.** Comparison of TCS quartiles (Q1, Q2, Q3 and Q4) with Ki67 levels at surgery (**A**) and ΔKi67 (**B**). *p* values were calculated using Mann Whitney test.

## Data Availability

The data for this study are available upon reasonable request.

## Abbreviations

BC: Breast cancer
BCS: Breast-conserving surgery
CR: Complete response
ER: Oestrogen receptor
HER2: Human epidermal growth factor receptor 2
mPEPI: Modified PEPI
MRI: Magnetic resonance imaging
NCT: Neoadjuvant chemotherapy
NET: Neoadjuvant endocrine therapy
NST: Neoadjuvant systemic treatment
path-TR: Pathological tumour response
path-TS: Pathological tumour size
pCR: Pathologic complete response
PD: Progressive disease
PEPI: Preoperative endocrine prognostic index
PgR: Progesterone receptor
PR: Partial response
rad-TR: Radiological tumour response
rad-TS: Radiological tumour size
RCB: Residual cancer burden
SD: Stable disease
SEM: Standard error of the mean
TCS: Tumour cellularity size
USS: Ultrasound scan

## References

[1] Burstein HJ (2020). Systemic therapy for estrogen receptor-positive, HER2-negative breast cancer. N Engl J Med.

[2] Spring LM, Gupta A, Reynolds KL, Gadd MA, Ellisen LW, Isakoff SJ (2016). Neoadjuvant endocrine therapy for estrogen receptor-positive breast cancer a systematic review and meta-analysis. JAMA Oncol.

[3] Sella T, Weiss A, Mittendorf EA, King TA, Pilewskie M, Giuliano AE (2021). Neoadjuvant endocrine therapy in clinical practice: a review. JAMA Oncol.

[4] Lerebours F, Pulido M, Fourme E, Debled M, Becette V, Bonnefoi H (2020). Predictive factors of 5-year relapse-free survival in HR+/HER2- breast cancer patients treated with neoadjuvant endocrine therapy: pooled analysis of two phase 2 trials. Br J Cancer.

[5] Cortazar P, Zhang L, Untch M, Mehta K, Costantino JP, Wolmark N (2014). Pathological complete response and long-term clinical benefit in breast cancer: the CTNeoBC pooled analysis. The Lancet.

[6] Eiermann W, Paepke S, Appfelstaedt J, Llombart-Cussac A, Eremin J, Vinholes J (2001). Preoperative treatment of postmenopausal breast cancer patients with letrozole: a randomized double-blind multicenter study. Ann Oncol.

[7] Cataliotti L, Buzdar AU, Noguchi S, Bines J, Takatsuka Y, Petrakova K (2006). Comparison of anastrozole versus tamoxifen as preoperative therapy in postmenopausal women with hormone receptor-positive breast cancer: the Pre-Operative ‘Arimidex’ Compared to Tamoxifen (PROACT) trial. Cancer.

[8] Smith IE, Dowsett M, Ebbs SR, Dixon JM, Skene A, Blohmer J-U (2005). Neoadjuvant treatment of postmenopausal breast cancer with anastrozole, tamoxifen, or both in combination: the Immediate Preoperative Anastrozole, Tamoxifen, or Combined with Tamoxifen (IMPACT) multicenter double-blind randomized trial. J Clin Oncol.

[9] Korde LA, Somerfield MR, Carey LA, Crews JR, Denduluri N, Hwang ES (2021). Neoadjuvant chemotherapy, endocrine therapy, and targeted therapy for breast cancer: ASCO guideline. J Clin Oncol.

[10] Martí C, Yébenes L, Oliver JM, Moreno E, Frías L, Berjón A (2022). The clinical impact of neoadjuvant endocrine treatment on luminal-like breast cancers and its prognostic significance: results from a single-institution prospective cohort study. Curr Oncol.

[11] Cardoso F, Kyriakides S, Ohno S, Penault-Llorca F, Poortmans P, Rubio IT (2019). Early breast cancer: ESMO Clinical Practice Guidelines for diagnosis, treatment and follow-up. Ann Oncol.

[12] Lerebours F, Rivera S, Mouret-Reynier MA, Alran S, Venat-Bouvet L, Kerbrat P (2016). Randomized phase 2 neoadjuvant trial evaluating anastrozole and fulvestrant efficacy for postmenopausal, estrogen receptor–positive, human epidermal growth factor receptor 2-negative breast cancer patients: Results of the UNICANCER CARMINA 02 french trial. Cancer.

[13] Skriver SK, Laenkholm AV, Rasmussen BB, Handler J, Grundtmann B, Tvedskov TF (2018). Neoadjuvant letrozole for postmenopausal estrogen receptor-positive, HER2-negative breast cancer patients, a study from the Danish Breast Cancer Cooperative Group (DBCG). Acta Oncol (Madr).

[14] Ueno T, Saji S, Masuda N, Kuroi K, Sato N, Takei H (2018). Impact of clinical response to neoadjuvant endocrine therapy on patient outcomes: a follow-up study of JFMC34-0601 multicentre prospective neoadjuvant endocrine trial. ESMO Open..

[15] Eisenhauer EA, Therasse P, Bogaerts J, Schwartz LH, Sargent D, Ford R (2009). New response evaluation criteria in solid tumours: revised RECIST guideline (version 1.1). Eur J Cancer.

[16] Ogston KN, Miller ID, Payne S, Hutcheon AW, Sarkar TK, Smith I (2003). A new histological grading system to assess response of breast cancers to primary chemotherapy: prognostic significance and survival. Breast.

[17] Sataloff DM, Mason BA, Prestipino AJ, Seinige UL, Lieber CP, Baloch Z (1995). Pathologic response to induction chemotherapy in locally advanced carcinoma of the breast: a determinant of outcome. J Am Coll Surg.

[18] Symmans WF, Wei C, Gould R, Yu X, Zhang Y, Liu M (2017). Long-term prognostic risk after neoadjuvant chemotherapy associated with residual cancer burden and breast cancer subtype. J Clin Oncol.

[19] Guarneri V, Dieci MV, Bisagni G, Frassoldati A, Bianchi GV, De Salvo GL (2019). De-escalated therapy for HR+/HER2+ breast cancer patients with Ki67 response after 2-week letrozole: results of the PerELISA neoadjuvant study. Ann Oncol.

[20] Smith I, Robertson J, Kilburn L, Wilcox M, Evans A, Holcombe C (2020). Long-term outcome and prognostic value of Ki67 after perioperative endocrine therapy in postmenopausal women with hormone-sensitive early breast cancer (POETIC): an open-label, multicentre, parallel-group, randomised, phase 3 trial. Lancet Oncol.

[21] Ellis MJ, Tao Y, Luo J, A’Hern R, Evans DB, Bhatnagar AS (2008). Outcome prediction for estrogen receptor-positive breast cancer based on postneoadjuvant endocrine therapy tumor characteristics. J Natl Cancer Inst.

[22] Suman VJ, Ellis MJ, Ma CX (2015). The ALTERNATE trial: assessing a biomarker driven strategy for the treatment of post-menopausal women with Er+/Her2- invasive breast cancer. Chin Clin Oncol.

[23] Ellis MJ, Suman VJ, Hoog J, Goncalves R, Sanati S, Creighton CJ (2017). Ki67 proliferation index as a tool for chemotherapy decisions during and after neoadjuvant aromatase inhibitor treatment of breast cancer: results from the American college of surgeons oncology group Z1031 trial (alliance). J Clin Oncol.

[24] Nielsen TO, Leung SCY, Rimm DL, Acs B (2021). Assessment of Ki67 in breast cancer: updated recommendations from the international Ki67 in Breast Cancer Working Group. J Natl Cancer Inst.

[25] Agostinetto E, Gligorov J, Piccart M (2022). Systemic therapy for early-stage breast cancer: learning from the past to build the future. Nat Rev Clin Oncol.

[26] Thomssen C, Balic M, Harbeck N, Gnant M (2021). St. Gallen/Vienna 2021: a brief summary of the consensus discussion on customizing therapies for women with early breast cancer. Breast Care.

[27] Denkert C, Seither F, Schneeweiss A, Link T, Blohmer JU, Just M (2021). Clinical and molecular characteristics of HER2-low-positive breast cancer: pooled analysis of individual patient data from four prospective, neoadjuvant clinical trials. Lancet Oncol.

[28] Kang S, Lee SH, Lee HJ, Jeong H, Jeong JH, Kim JE (2022). Pathological complete response, long-term outcomes, and recurrence patterns in HER2-low versus HER2-zero breast cancer after neoadjuvant chemotherapy. Eur J Cancer.

[29] Heil J, Kuerer HM, Pfob A, Rauch G, Sinn HP, Golatta M (2020). Eliminating the breast cancer surgery paradigm after neoadjuvant systemic therapy: current evidence and future challenges. Ann Oncol.

[30] Hortobagyi GN, Connolly JL, Orsi CJD, Edge SB, Mittendorf EA, Rugo HS, et al. AJCC Cancer Staging Manual 8th Edition. Definitions. (2020)

[31] Dowsett M, Nielsen TO, A’Hern R, Bartlett J, Coombes RC, Cuzick J (2011). Assessment of Ki67 in Breast Cancer: recommendations from the international Ki67 in breast cancer working Group. J Natl Cancer Inst.

[32] Hammond MEH, Hayes DF, Dowsett M, Allred DC, Hagerty KL, Badve S (2010). American Society of Clinical Oncology/College Of American Pathologists guideline recommendations for immunohistochemical testing of estrogen and progesterone receptors in breast cancer. J Clin Oncol.

[33] Skriver SK, Jensen MB, Knoop AS, Ejlertsen B, Laenkholm AV (2020). Tumour-infiltrating lymphocytes and response to neoadjuvant letrozole in patients with early oestrogen receptor-positive breast cancer: analysis from a nationwide phase II DBCG trial. Breast Cancer Res.

[34] Martí C, Sánchez-méndez JI (2021). The present and future of neoadjuvant endocrine therapy for breast cancer treatment. Cancers (Basel)..

[35] Reis J, Lindstrøm JC, Boavida J, Gjesdal KI, Park D, Bahrami N (2020). Accuracy of breast MRI in patients receiving neoadjuvant endocrine therapy: comprehensive imaging analysis and correlation with clinical and pathological assessments. Breast Cancer Res Treat.

[36] Fowler AM, Mankoff DA, Joe BN (2017). Imaging neoadjuvant therapy response in breast cancer. Radiology.

