# Supplementary material for: A prospective study on tumour response assessment methods after neoadjuvant endocrine therapy in early oestrogen receptor-positive breast cancer
Source: Breast Cancer Res. 2024 Jan 3;26:3. doi: 10.1186/s13058-023-01756-8 (PMC10765775; doi:10.1186/s13058-023-01756-8)

Supplementary material online

Supplementary figure legends:

Figure S1. Changes in tumour levels of: Ki67 (A), Oestrogen receptor (B) and Progesterone receptor (C) before and after NET.

Figure S2. Association between tumour cellularity size (A), tumour cellularity (B) and pathological tumour size (C) with Ki67 levels at surgery. Spearman correlation coefficients (rho) and *p* values are shown.

Figure S3. Comparison of TCS quartiles (Q1, Q2, Q3 and Q4) with Ki67 levels at surgery (A) and ∆Ki67 (B). *p* values were calculated using Mann Whitney test.

Supplementary Figure S1

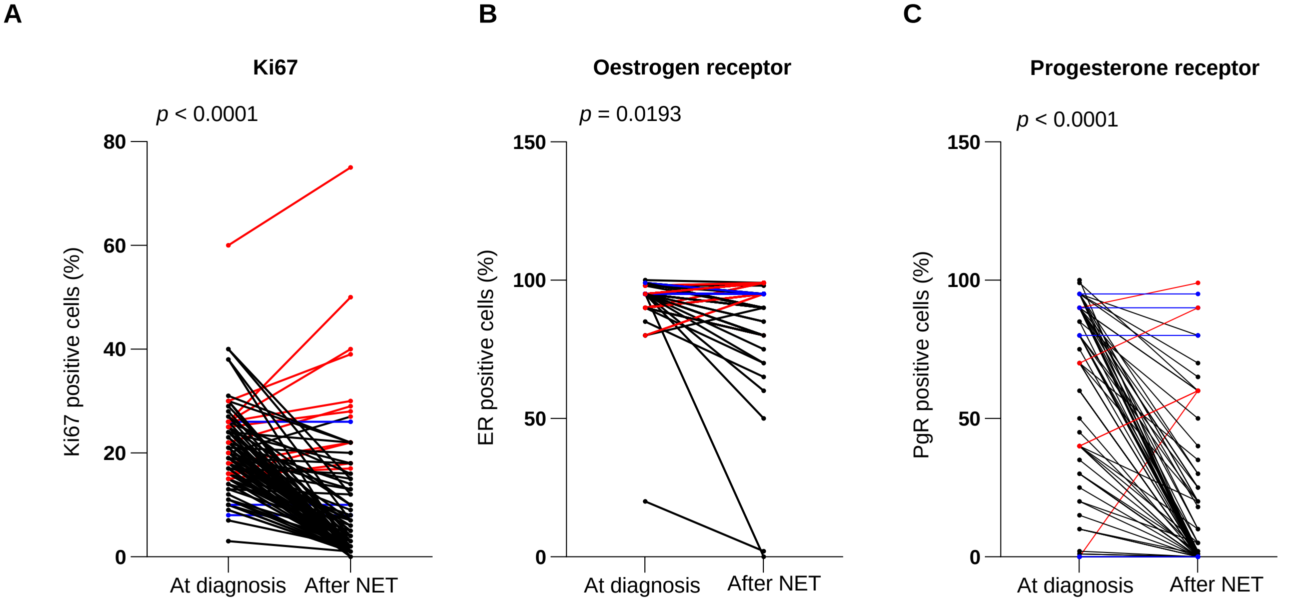

Positive cells (%): **mean** (range)	At diagnosis	After NET
A Ki67	**20** (3-60)	**9** (0-75)
B Oestrogen receptor	**94** (20-100)	**90** (0-99)
C Progesterone receptor	**63** (0-100)	**14** (0-99)

Change between at diagnosis and after NET:
Decrease; Increase; No change

Supplementary Figure S2

A

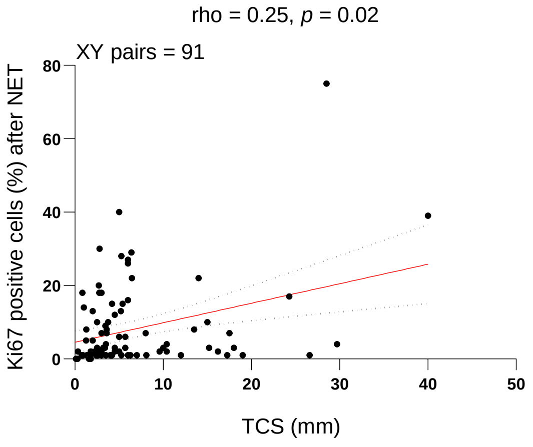

B

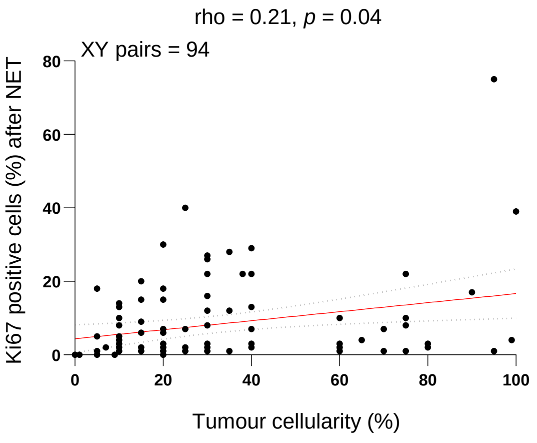

C

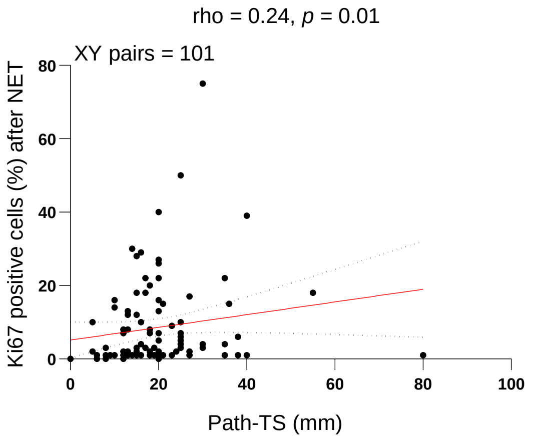

Supplementary Figure S3

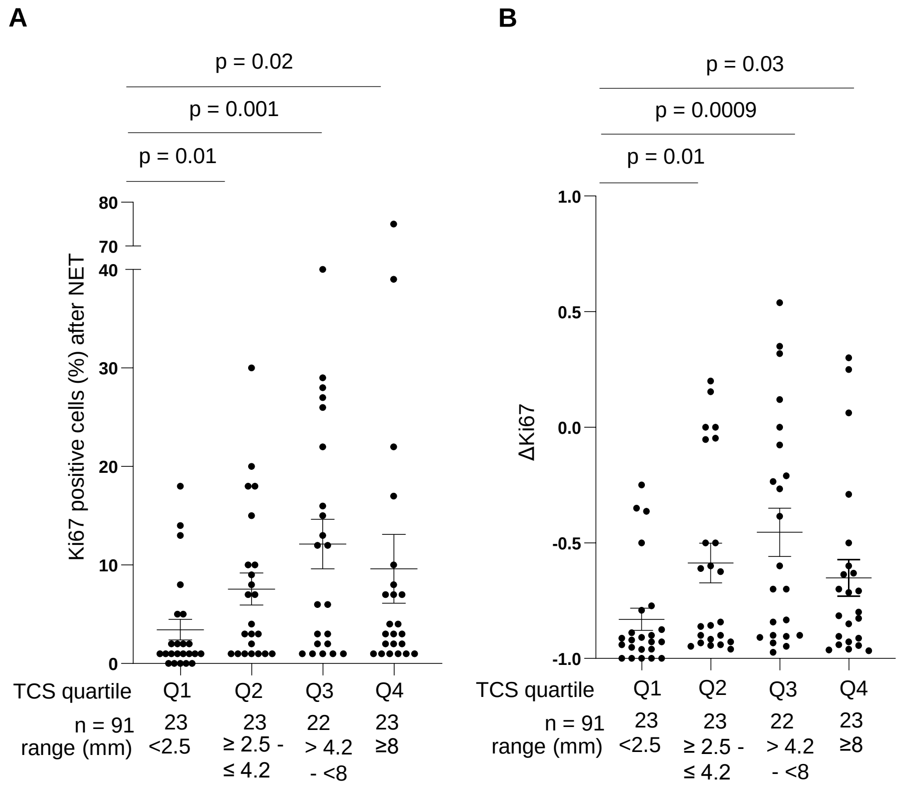

Supplement: Supplementary file 1 — Additional file 1. Figure S1. Changes in tumour levels of: Ki67 (A), Oestrogen receptor (B) and Progesterone receptor (C) before and after NET. Figure S2. Association between tumour cellularity size (A), tumour cellularity (B) and pathological tumour size (C) with Ki67 levels at surgery. Spearman correlation coefficients (rho) and p values are shown. Figure S3. Comparison of TCS quartiles (Q1, Q2, Q3 and Q4) with Ki67 levels at surgery (A) and ΔKi67 (B). p values were calculated using Mann Whitney test. [file 13058_2023_1756_MOESM1_ESM.pdf]
